# The Pathway Relationship Between Physical Activity Levels and Depressive Symptoms in University Students Mediated by Cognitive Flexibility

**DOI:** 10.1002/brb3.70285

**Published:** 2025-01-21

**Authors:** Fen Yu, Shuqi Jia, Qin Liu, Zhaohui Guo, Sen Li, Xing Wang, Pan Li

**Affiliations:** ^1^ School of Physical Education Shanghai University of Sport Shanghai China; ^2^ School of Physical Education and Health Shanghai Lixin University of Accounting and Finance Shanghai China

**Keywords:** cognitive flexibility, depressive symptoms, physical activity level

## Abstract

**Objective:**

To explore the mediating pathway of cognitive flexibility in the relationship between physical activity and depressive symptoms among university students.

**Methods:**

A cross‐sectional design was used to survey 2537 university students using the Physical Activity Rating Scale‐3, Patients’ Health Questionnaire, and Cognitive Flexibility Inventory. Data analysis was conducted using independent samples *t*‐test, chi‐square test, correlation analysis, one‐way ANOVA, and mediation analysis.

**Results:**

A statistically significant negative correlation exists between the intensity of physical activity and depression symptom scores (*r* = −0.104, *p *< 0.01). The intensity of physical activity demonstrates a statistically significant positive correlation with controllability (*r* = 0.109, *p *< 0.01). A marked negative correlation is observed between depressive symptoms and controllability scores (*r* = −0.367, *p *< 0.01). The total effect of physical activity intensity on depressive symptoms was quantified as −0.3542 (95% CI: −0.5439 to −0.1645). The direct effect was found to be ‐0.2199 (95% CI: ‐0.3981, ‐0.0417), while the mediating effect of controllability was calculated to be −0.1343 (95% CI: −0.2145 to −0.0630).

**Conclusion:**

Increased engagement in physical activity among university students is associated with a reduction in their depressive symptom scores. Controllability serves as a mediating factor in the relationship between physical activity and depressive symptoms among university students.

## Graphical abstract

To explore the mediating pathway of cognitive flexibility in the relationship between physical activity and depressive symptoms among university students. Controllability serves as a mediating factor in the relationship between physical activity intensity and depressive symptoms among university students.

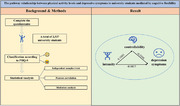

## 1 Introduction

Depressive symptoms refer to a negative emotional disorder that occurs when an individual is unable to cope with stressful life events (Yin et al. 2024). In China, the lifetime prevalence of depression is 3.4%, and the detection rate of depressive symptoms among university students is as high as 20.8%, showing a gradual upward trend (Y. Chen, Zhang, and Yu 2022). Individuals with depressive symptoms, under the influence of persistent depressive mood, may experience sleep and eating problems, feel very fatigued and lacking in energy, and, in severe cases, may even exhibit suicidal behavior (H. Liu and Wang 2017; C. Liu et al. 2019). Depression has become a significant global public health issue, and it is urgent to improve depressive symptoms among university students.

Decreased cognitive flexibility is one of the main characteristics of university students with depressive symptoms (Zong et al. 2010), primarily manifested in the dimensions of controllability and alternative options. Research has found that cognitive flexibility is an important factor influencing the development of depressive symptoms (Zhou et al. 2021; Gao et al. 2020). Individuals with higher levels of cognitive flexibility are likely to have a lower probability of developing depressive symptoms (Jung 2019). Individuals with lower cognitive flexibility have difficulty adapting to new environments and challenges, are prone to negative emotions such as self‐denial, and often become trapped in these negative emotions, ultimately leading to the development of depressive and other negative emotions (Gabrys et al. 2018). Furthermore, previous studies have found that one dimension of cognitive flexibility, known as controllability, involves the rapid processing of new information to ascertain the next appropriate action (X. Wang et al. 2024). This may reduce the negative evaluation of stressful situations by individuals with depressive symptoms. The dimension of controllability seems to be more closely related to the generation of negative emotions.

Physical activity has a strong antidepressant effect (Fang and Guo 2019). The higher the score on the Physical Activity Rating Scale, the lower the detection rate of depressive symptoms (Zhang et al. 2020). Research has found that during physical activity, there is increased activation in the bilateral superior frontal gyrus, bilateral middle frontal gyrus, and bilateral superior parietal lobule. Additionally, the activation levels in the prefrontal and parietal regions, which are highly related to cognitive flexibility, also significantly increase (S. Liu et al. 2020). Positive experiences gained during physical activity can help individuals combat depressive symptoms (Li, Jia, and Zuo 2015). Concurrently, some researchers suggest that various components of physical activity significantly influence cognitive flexibility (Gai et al. 2021). Specifically, the intensity component of physical activity may differentially impact the activation level of individual cognitive abilities, thereby influencing the cognitive benefits derived from physical activity (de Greeff et al. 2018). Physical activity can alleviate brain plasticity degradation by improving the reduced volume, structural degradation, and functional disorders of the hippocampus, prefrontal cortex, and anterior cingulate cortex (X. Chen et al. 2024). It enhances activation levels and functional connectivity between brain regions, regulates the expression of brain‐derived neurotrophic factor (BDNF) (Heyman et al. 2012), and the synthesis and release of monoamine neurotransmitters (Lin and Kuo 2013). Additionally, it modulates abnormalities in the hypothalamic–pituitary–adrenal axis, oxidative stress levels (S. Wang et al. 2022), and gut microbiota balance in the body (X. Liu et al. 2021). Consequently, these changes improve cognitive flexibility levels, thereby exerting an antidepressant effect.

Reviewing previous research findings reveals that regular physical activity plays a positive role in maintaining good levels of cognitive flexibility and alleviating depressive symptoms. Is there a close relationship between physical activity, depressive symptoms, and cognitive flexibility? What is the mediating pathway of cognitive flexibility between physical activity and depressive symptoms? Clarifying this relationship may provide new perspectives for alleviating depressive symptoms among university students. This study aims to employ a cross‐sectional design to gather data on university students’ levels of physical activity, depressive symptoms, and cognitive flexibility. The proposed hypotheses are as follows: H1: Physical activity levels are correlated with depressive symptoms. H2: Different components of physical activity have varying effects on depressive symptoms. H3: The relationship between physical activity intensity and depressive symptoms is mediated by controllability.

## 2 Methods

### 2.1 Participant Recruitment

**FIGURE 1:**
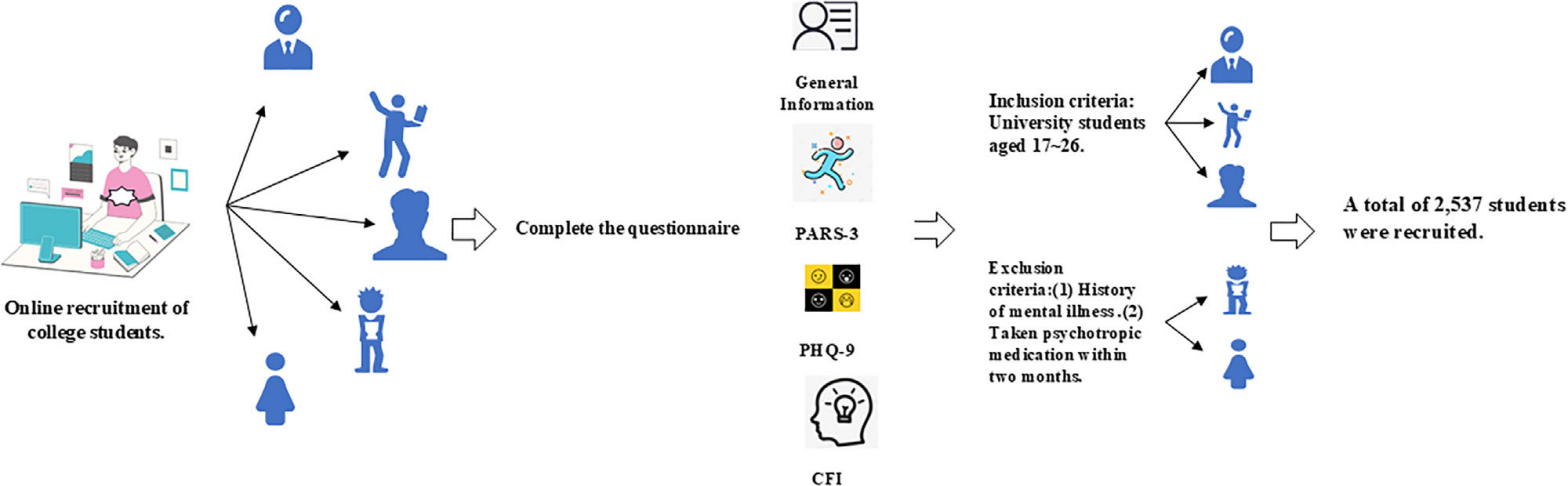
Participant recruitment process.

The study adhered to the principle of voluntary participation and employed a cluster sampling method. A total of 2663 university students were randomly selected from 50 classes across 8 colleges in Songjiang District, Shanghai. Inclusion criteria were university students aged 17–26 years. Exclusion criteria included: (1) history of mental illness and (2) use of psychiatric medication within the past 2 months. This study adheres to the ethical requirements of the latest version of the Declaration of Helsinki. It has been reviewed and approved by the Ethics Committee of Shanghai University of Sport (Ethics Registration Number: 102772023RT075). With the consent of the relevant sports department authorities, trained researchers posted recruitment posters and distributed recruitment leaflets to recruit participants. For individuals meeting the recruitment criteria, the possibility of their inclusion was preliminarily assessed based on inclusion and exclusion criteria. Participants were informed about the study's purpose and content and signed informed consent forms, indicating their voluntary participation. The recruitment and screening period was from October 2023 to April 2024. The participant recruitment process is illustrated in Figure 1.

### 2.2 Measurement Tools

#### 2.2.1 General Information Questionnaire

Including basic information such as the participants’ age, BMI, gender, only‐child status, household registration location, single‐parent status, alcohol consumption, and smoking habits.

#### 2.2.2 Physical Activity Rating Scale‐3

Based on the Chinese version of the Physical Activity Rating Scale (PARS) revised by Liang (1994), this study assesses participants’ physical activity levels by considering the intensity, duration, and frequency of physical activity. The physical activity level score is calculated as the product of exercise intensity, duration, and frequency. The Cronbach's *α* coefficient for this scale is 0.856, and the test–retest reliability is 0.82 (X. Liu et al. 1995).

#### 2.2.3 Patients’ Health Questionnaire

The questionnaire includes nine items, with response options of “not at all,” “several days,” “more than half the days,” and “nearly every day,” scored as 0, 1, 2, and 3, respectively (Kroenke, Spitzer, and Williams 2001). The total score ranges from 0 to 27, with higher scores indicating more severe depressive symptoms. The Patients’ Health Questionnaire (PHQ‐9) has demonstrated good reliability and validity, with a Cronbach's *α* coefficient of 0.84 (Vrublevska, Trapencieris, and Rancans 2017).

#### 2.2.4 Cognitive Flexibility Inventory

This study utilized the Cognitive Flexibility Inventory (CFI) developed by Dennis and Vander Wal, with the Chinese version translated by domestic scholar Y. Wang et al. (2016), to assess patients’ cognitive flexibility. The questionnaire consists of 20 items and includes two dimensions: “alternatives” and “control.” “Alternatives” refers to the ability to adopt multiple approaches to deal with difficulties, while “control” indicates the individual's awareness that the challenging situations they face are controllable (Y. Wang et al. 2016). Each item is rated on a 5‐point Likert scale, with a total score ranging from 20 to 100; higher scores indicate better cognitive flexibility. The CFI has demonstrated good reliability and validity, with a Cronbach's *α* coefficient of 0.88.

### 2.3 Data Collection

Participants completed an online survey that included a basic information form, the Physical Activity Rating Scale, the Patient Health Questionnaire, and the CFI. Before filling out the questionnaires, the investigators explained each item, clarified that the collected data would be used solely for scientific research, and informed the participants of their right to complete the survey independently and truthfully, as well as their right to withdraw at any time. During the survey, participants were reminded to answer carefully. Upon completion, the investigators checked for patterned responses, completion times less than 3 min, and questionnaires with more than one‐third of the items unanswered. A total of 126 invalid questionnaires were excluded, including 39 with patterned responses, 41 with completion times less than 3 min, and 46 with more than one‐third of the items unanswered. Finally, 2537 questionnaires were included in the analysis, resulting in an effective response rate of 95.27%.

### 2.4 Statistical Analysis

Statistical analyses were conducted using SPSS version 29.0. An independent samples *t*‐test and chi‐square test were employed to compare demographic characteristics between university students with and without depressive symptoms. Correlation analysis was used to explore the relationships among physical activity intensity, depressive symptoms, and cognitive flexibility. One‐way ANOVA was performed to compare the differences in scores for depressive symptoms and cognitive flexibility across different levels of physical activity intensity. Post hoc tests were conducted to compare the differences in scores for depressive symptoms and cognitive flexibility between low‐, moderate‐, and high‐intensity physical activity groups. The Process 3.5 macro was used to analyze the mediating effect of cognitive flexibility, serving as a mediator between physical activity intensity and depressive symptoms.

## 3 Results

### 3.1 Common Method Variance

In this study, all items were completed by the participants themselves, which may result in common method variance. To test for the presence of common method bias in this study, Harman's single‐factor test was conducted. The first factor accounted for 32.52% of the total variance without rotation, which is less than 40% threshold. This indicates that there is no serious common method bias in this study, allowing us to proceed with further data analysis.

### 3.2 Demographic Information

A total of 2537 participants were included in the study, with a depression detection rate of 48.76% in the overall sample. The age of the participants in the groups with and without depressive symptoms was (19.232 ± 1.409) years and (19.313 ± 2.449) years, respectively; the BMI was (22.361 ± 5.156) kg/m^2^ and (22.107 ± 4.899) kg/m^2^, respectively. The number of males was 513 (41.5%) and 635 (48.8%), and the number of females was 724 (58.5%) and 665 (51.2%), respectively. The number of only children was 428 (34.6%) and 488 (37.5%); urban household registration accounted for 526 (42.5%) and 544 (41.8%); single‐parent families accounted for 113 (9.1%) and 114 (8.8%); those with a drinking habit accounted for 296 (23.9%) and 258 (19.8%); and those with a smoking habit accounted for 83 (6.8%) and 107 (8.3%). Regarding overall social activity, 14.0% reported “never,” 79.2% reported “1–3 times per week,” 5% reported “4–6 times per week,” and 1.8% reported “daily.” Regarding overall family situation, 65.6% reported “harmonious,” 30.7% reported “occasional conflicts,” 3.2% reported “frequent conflicts,” and 0.6% reported “severe conflicts.” Regarding overall interpersonal relationships, 31.7% reported “good,” 44.8% reported “fairly good,” 22.2% reported “average,” 0.8% reported “fairly poor,” and 0.5% reported “poor.” Regarding overall academic difficulty, 8.4% reported “easy,” 16.6% reported “fairly easy,” 58.1% reported “moderate,” 13.8% reported “fairly difficult,” and 3.1% reported “difficult.” Detailed demographic information is shown in Table 1.

**TABLE 1:** Demographic information.

Variables	Overall (*n* = 2537)	With depressive symptoms (*n* = 1237)	Without depressive symptoms (*n* = 1300)	Difference test (*t*/*χ* ^2^)
Age (years)	22.231 ± 5.026	19.229 ± 1.409	19.314 ± 2.449	*t* = 1.065, *p* = 0.287
BMI/(kg/m^2^)	19.272 ± 2.010	22.361 ± 5.156	22.107 ± 4.899	*t* = −1.270, *p* = 0.204
Gender (%)				*χ* ^2 ^= 13.915, *p *< 0.001
Male	1148 (45.3%)	513 (41.5%)	635 (48.8%)	
Female	1389 (54.7%)	724 (58.5%)	665 (51.2%)	
Only child (%)				*χ* ^2 ^= 2.373, *p* = 0.126
Yes	916 (36.1%)	428 (34.6%)	488 (37.5%)	
No	1621 (63.9%)	809 (65.4%)	812 (62.5%)	
Household registration (%)				*χ* ^2 ^= 0.119, *p* = 0.748
Urban	1070 (42.2%)	526 (42.5%)	544 (41.8%)	
Rural	1467 (57.8%)	711 (57.5%)	756 (58.2%)	
Single‐parent family (%)				*χ* ^2^ = 0.104, *p* = 0.781
Yes	227 (8.9%)	113 (9.1%)	114 (8.8%)	
No	2310 (91.1%)	1124 (90.9%)	1186 (91.2%)	
Drinking habit (%)				*χ* ^2^ * = *6.190, *p = *0.014
Yes	554 (21.8%)	296 (23.9%)	258 (19.8%)	
No	1983 (78.2%)	941 (76.1%)	1042 (80.2%)	
Smoking habit (%)				*χ* ^2^ * = *1.888, *p = *0.176
Yes	191 (7.5%)	84 (6.8%)	107 (8.2%)	
No	2346 (92.5%)	1153 (93.2%)	1193 (91.8%)	
Social activities (%)				*χ* ^2^ * = *26.647, *p* < 0.001
Never	356 (14.0%)	213 (17.2%)	143 (11.0%)	
1–3 times per week	2009 (79.2%)	954 (77.1%)	1055 (81.5%)	
4–6 times per week	127 (5%)	57 (4.6%)	70 (5.4%)	
Daily	45 (1.8%)	13 (1.1%)	32 (2.5%)	
Family situation (%)				*χ* ^2^ * = *86.353, *p* < 0.001
Harmonious	1664 (65.6%)	707 (56.8%)	961 (73.9%)	
Occasional conflicts	779 (30.7%)	467 (37.8%)	312 (24.0%)	
Frequent conflicts	80 (3.2%)	57 (4.6%)	23 (1.8%)	
Severe conflicts	14 (0.6%)	10 (0.8%)	4 (0.3%)	
Interpersonal relationships (%)				*χ* ^2^ * = *182.456, *p* < 0.001
Good	804 (31.7%)	253 (20.5%)	551 (42.4%)	
Fairly good	1137 (44.8%)	585 (47.3%)	552 (42.5%)	
Average	563 (22.2%)	372 (30.1%)	191 (14.7%)	
Fairly poor	20 (0.8%)	15 (1.2%)	5 (0.4%)	
Poor	13 (0.5%)	12 (1.0%)	1 (0.1%)	
Academic difficulty (%)				*χ* ^2^ * = *79.637, *p *< 0.001
Easy	213 (8.4%)	69 (5.6%)	144 (11.1%)	
Fairly easy	422 (16.6%)	175 (14.1%)	247 (19.0%)	
Moderate	1473 (58.1%)	713 (57.6%)	760 (58.5%)	
Fairly difficult	351 (13.8%)	233 (18.8%)	118 (9.1%)	
Difficult	78 (3.1%)	47 (3.8%)	31 (2.4%)	

### 3.3 Differences in Cognitive Flexibility Between University Students With and Without Depressive Symptoms

**FIGURE 2:**
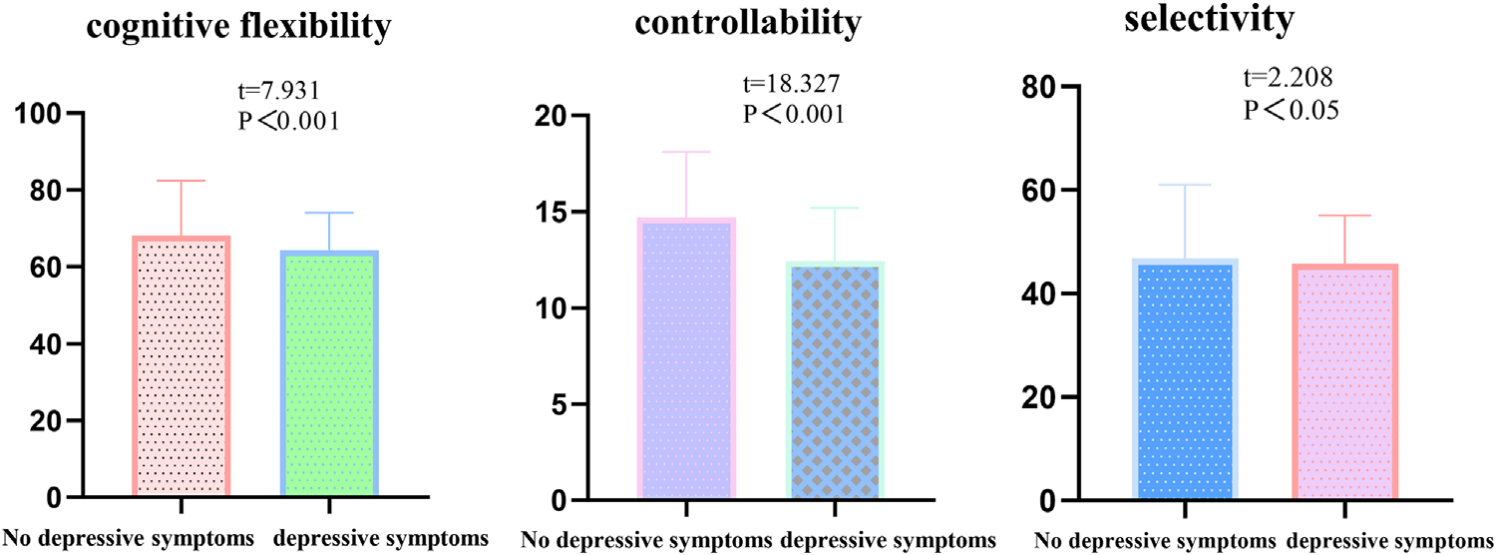
Differences in cognitive flexibility between university students with and without depressive symptoms.

As shown in Table 2 and Figure 2, there are significant differences in cognitive flexibility between university students with and without depressive symptoms (*t* = 7.931, *p *< 0.001). Specifically, there are significant differences in controllability (*t* = 18.327, *p *< 0.001) and selectivity (*t* = 2.208, *p *< 0.05).

**TABLE 2:** Differences in cognitive flexibility between university students with and without depressive symptoms.

Variables	With depressive symptoms (*n* = 1237)	Without depressive symptoms (*n* = 1300)	*t*‐value	*p*‐value
Cognitive flexibility	64.329 ± 9.707	68.182 ± 14.221	7.931	< 0.001
Controllability	12.407 ± 2.799	14.689 ± 3.425	18.327	< 0.001
Selectivity	45.707 ± 9.378	46.769 ± 4.224	2.208	< 0.05

### 3.4 Correlation Between Depressive Symptom Scores, Cognitive Flexibility, and Physical Activity Levels in University Students

**FIGURE 3:**
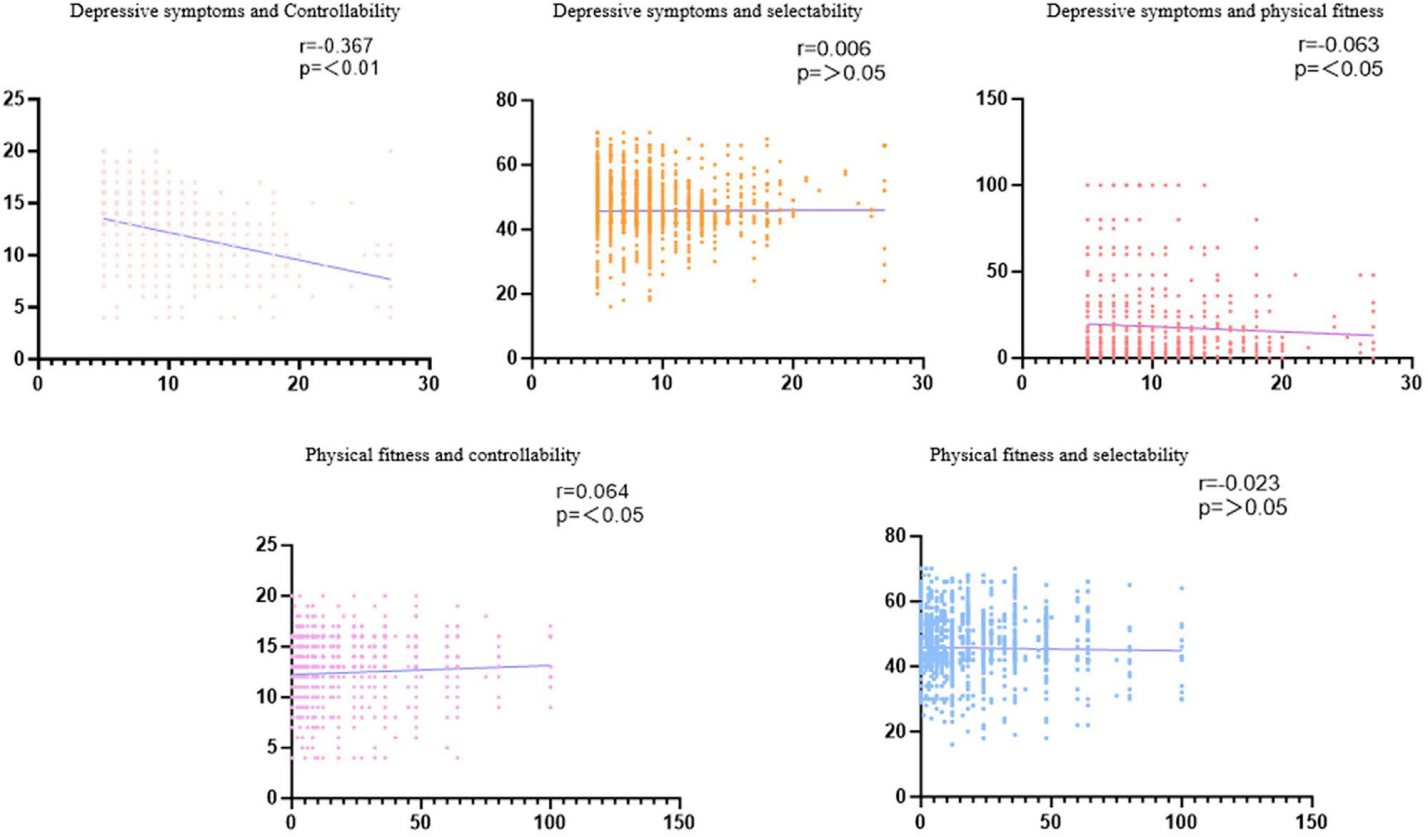
Correlation between physical activity levels, cognitive flexibility, and depressive symptoms.

Table 3 and Figure 3 demonstrate that the level of physical activity has a weak yet statistically significant negative correlation with depressive symptom scores (*r* = −0.063, *p *< 0.05). Furthermore, a statistically significant positive correlation exists between the level of physical activity and controllability scores (*r* = 0.064, *p *< 0.05). The intensity of physical activity is negatively correlated with depressive symptom scores (*r* = −0.104, *p *< 0.01) and is positively correlated with controllability scores (*r* = 0.109, *p *< 0.01). Moreover, depressive symptoms exhibit a significant negative correlation with controllability scores (*r* = −0.367, *p *< 0.01).

**TABLE 3:** Correlation analysis of physical activity levels and cognitive flexibility among university students with depressive symptoms.

Pearson correlation	Depressive symptoms	Controllability	Selectivity	Physical activity level	Intensity	Frequency	Duration
Depressive symptoms	1						
Controllability	−0.367^**^	1					
Selectivity	0.006	−0.257^**^	1				
Physical activity level	−0.063^*^	0.064^*^	−0.023	1			
Intensity	−0.104^**^	0.109^**^	−0.021	0.113^**^	1		
Frequency	−0.035	0.060^*^	0.107^**^	0.126^**^	0.182^**^	1	
Duration	−0.047	0.091^**^	−0.046	0.080^**^	0.477^**^	0.214^**^	1

* *p *< 0.05.

** *p *< 0.01.

One‐way ANOVA was used to test the differences in depressive symptom scores and controllability among university students with depressive symptoms at different levels of physical activity intensity and frequency. The results showed significant differences in depressive symptom scores (*F* = 7.628, *p *< 0.001) and controllability scores (*F* = 4.544, *p* < 0.05) among students with depressive symptoms across different levels of activity intensity. Post hoc multiple comparisons indicated that the depressive symptom scores of the low‐intensity and moderate‐intensity groups were significantly higher than those of the high‐intensity group (*p *< 0.001), with no significant difference between the low‐intensity and moderate‐intensity groups. The controllability scores of the moderate‐intensity and high‐intensity groups were significantly higher than those of the low‐intensity group (*p* < 0.05), with no significant difference between the moderate‐intensity and high‐intensity groups (Table 4).

**TABLE 4:** Differences in depressive symptoms and cognitive flexibility scores across different intensities of physical activity.

Variables	Intensity of physical activity			Difference test		Post hoc multiple comparisons		
	Low intensity (*n* = 439)	Moderate intensity (*n* = 594)	High intensity (*n* = 204)	*F*‐value	*p*‐value	Low vs. moderate	Low vs. high	Moderate vs. high
Depressive symptoms	9.467 ± 4.067	9.249 ± 4.060	8.216 ± 2.647	7.628	< 0.001	0.371	< 0.001	< 0.01
Controllability	12.107 ± 2.809	12.507 ± 2.801	12.760 ± 2.721	4.544	< 0.05	< 0.05	< 0.01	0.264

### 3.5 The Mediating Role of Cognitive Flexibility in the Improvement of Depressive Symptoms Through Physical Activity

**FIGURE 4:**
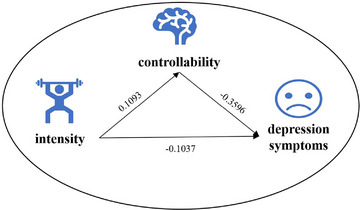
Mediating role of controllability in the pathway between activity intensity and depressive symptoms. *Note*: Path coefficients are standardized values.

The mediation effect test with physical activity intensity as the independent variable, controllability as the mediator variable, and depressive symptoms as the dependent variable. The results show that intensity predicts depressive symptoms (*R* ^2^ = 0.0107, *F* = 13.4149, *β* = −0.1037, *t* = −3.6626, *p* < 0.001), intensity predicts controllability (*R* ^2^ = 0.0119, *F* = 14.9246, *β* = 0.1093, *t* = 3.8632, *p* < 0.001), and both intensity and controllability jointly predict the regression coefficients of depressive symptoms (*R* ^2^ = 0.1385, *F* = 99.2125; *β* = ‐0.0644, *t* = −2.4215, *p *< 0.05; *β* = −0.3596, *t* = −13.5290, *p *< 0.001) (Tables 5 and 6; Figure 4).

**TABLE 5:** Regression analysis of the relationships among variables in the mediation model (*N* = 1237).

Variables	Model 1		Model 2		Model 3	
	*β*	*t*	*β*	*t*	*β*	*t*
Intensity	−0.1037	−3.6626^***^	0.1093	3.8632^***^	−0.0644	−2.4215^*^
Controllability					−0.3596	−13.5290^***^
*R* ^2^	0.0107		0.0119		0.1385	
*F*	13.4149^***^		14.9246^***^		99.2125^***^	

*Note*: (1) Standardized values for all variables were used in the regression equation. (2) Model 1—Intensity predicts depressive symptoms; Model 2—Intensity predicts controllability; Model 3—Intensity and controllability jointly predict depressive symptoms.

* *p *< 0.05.

^**^ *p* < 0.01.

*** *p *< 0.001.

**TABLE 6:** Analysis of the mediating effect of controllability on the relationship between activity intensity and depressive symptoms.

	Effect size	Standard error	Bootstrap 95% CI		Proportion of total effect
			Lower limit	Upper limit	
Total effect	−0.3542	0.0967	−0.5439	−0.1645	
Direct effect	−0.2199	−0.0908	−0.3981	−0.0417	62.08%
Indirect effect	−0.1343	0.0388	−0.2145	−0.0630	37.92%

## 4 Discussion

The results of this study indicate that physical activity levels are inversely related to depressive symptoms and directly related to controllability. Additionally, depressive symptoms are inversely related to controllability. These findings are consistent with previous studies and support Hypothesis 1. These findings are consistent with previous research. He and Xu (2002) found a negative correlation between physical activity levels and depressive symptoms among university students. Wu and Fu (2024) discovered a significant positive correlation between physical activity and cognitive flexibility. The study by Ang et al. (2020) revealed a negative correlation between cognitive flexibility and depressive symptoms. In contrast to previous studies that have demonstrated a negative correlation between physical activity and depressive symptoms (Xiang et al. 2020; Farren et al. 2018; Wei et al. 2024), our study finds a weaker correlation. This may be attributed to the study population being college students, who often encounter a variety of internal and external challenges such as academic pressures, social interactions, family dynamics, teacher–student relationships, and difficulties in adapting to social environments. These complex factors can lead to increased somatic symptoms among college students, particularly those with depressive tendencies, which are reflected in their daily activities. Consequently, the influence of physical activity on depressive symptoms may be diminished. This aligns with the initial premise of our study that interactions among various components of physical activity can reduce the overall correlation between physical activity scores and depressive symptoms. These findings may reflect the unique characteristics of university students and the role of physical activity intensity. Furthermore, while this study utilized the PHQ‐9 to assess depressive symptoms, previous studies often employed the Self‐Rating Depression Scale or the 20‐item Center for Epidemiologic Studies Depression Scale for Children, leading to potential inconsistencies. Additionally, as the study was conducted post–COVID‐19 pandemic, the transitioning back to normalcy for many in China, coupled with the relatively relaxed lifestyles of university students compared to strict home quarantines, may have also influenced the results.

The results of this study suggest that the intensity of physical activity can significantly impact depressive symptoms, thus providing support for Hypothesis 2. A review focusing on individuals experiencing nonclinical depressive symptoms demonstrated that diverse intensities of physical activity produce distinct influences on depressive symptoms (Paolucci et al. 2018). Nonetheless, studies reveal that cerebral blood flow enhancement may differ across various intensity levels, where higher intensities could more effectively reduce vascular resistance and enhance cerebral blood flow (Singh et al. 2023). Furthermore, this study revealed a more significant correlation between the intensity of physical activity and the aspect of controllability. The hemoneurological hypothesis proposes that an increase in blood flow could potentially enhance the efficiency of local nerves that participate in information processing (Moore and Cao 2008). Nevertheless, research suggests variability in the enhancement of cerebral blood flow across different intensity levels, where higher intensities might effectively reduce vascular resistance and substantially increase cerebral blood flow (Tari et al. 2020). This might lead to more effective enhancements in local neural network efficiency, thereby providing cognitive benefits and improved controllability for individuals engaged in physical activities.

Further mediation analyses in this study revealed that among college students exhibiting depressive symptoms, the intensity of physical activity exerted a direct effect on those symptoms, accounting for 62.08%. High‐intensity physical activity can improve frontal alpha asymmetry, inducing lower negative emotions in students with depressive symptoms (Qiu et al. 2022). Moderate‐intensity physical activity can reduce tumor necrosis factor‐alpha (TNF‐α) and alleviate the severity of depressive symptoms (Paolucci et al. 2018). Low‐intensity physical activity can moderately improve depressive symptoms in individuals with poor physical condition (Conn 2010). The frequency and duration of physical activity among university students with depressive symptoms are relatively low, and whether there is a nonlinear relationship between depressive symptoms and the frequency and duration of physical activity requires further investigation. Additionally, this study identified that only the controllability aspect of cognitive flexibility serves as a mediator in the relationship between physical activity intensity and depressive symptoms, confirming Hypothesis 3. This may provide new insights, suggesting that depressive symptoms are more closely related to the level of controllability. Evidence shows that lower levels of controllability may be associated with higher levels of depressive symptoms (Zhong and Li 2004). Similar studies have found that controllability mediates the relationship between childhood psychological abuse and depressive symptoms among university students (B. Wang and Liu 2017). This could be because individuals with better controllability tend to process information with a positive attitude (Gan et al. 2007), thereby alleviating the emotional regulation difficulties and rumination in individuals with depressive symptoms (Zong et al. 2010; Lemoult and Gotlib 2019), making them less likely to experience physical and mental fatigue, and thus reducing the occurrence of depressive symptoms. Additionally, some studies indicate that the regulation of cognitive flexibility is more closely linked to changes in intensity (Ekkekakis 2003; Ekkekakis, Hall, and Petruzzello 2005). Physical activity may regulate the level of controllability by altering activity intensity, thereby affecting body self‐efficacy and sensory cues from respiratory muscles (Du, Ren, and Wang 2013; H. Wang et al. 2013; Qi 2013).

This study also found that selectivity did not mediate the relationship between physical activity and depressive symptoms, which is inconsistent with previous research (Fresco, Rytwinski, and Craighead 2007). A possible reason for this discrepancy is that this study assessed depressive symptoms in university students using the PHQ‐9, which includes nine items mostly related to difficult situations. Research has shown that controllability in difficult situations is more strongly associated with depressive symptoms (Fresco, Williams, and Nugent 2006).

This study also has certain limitations. The low level of correlation between the total score of physical activity level and depressive symptoms in the present study may have an impact on the conclusions, which can be further justified in the future by using longitudinal studies. Additionally, excluding individuals with missing key data on physical activity, depressive symptoms, and cognitive flexibility may introduce selection bias.

## Author Contributions

**Fen Yu**: writing–original draft, writing–review and editing, software, visualization, data curation. **Shuqi Jia**: methodology, data curation, visualization, writing–review and editing. **Qin Liu**: project administration. **Zhaohui Guo**: project administration. **Sen Li**: resources. **Xing Wang**: data curation. **Pan Li**: supervision.

## Ethics Statement

This study has been approved by the Ethics Committee of Shanghai University of Sport (102772023RT075).

## Conflicts of Interest

The authors declare no conflicts of interest.

### Peer Review

The peer review history for this article is available at https://publons.com/publon/10.1002/brb3.70285.

## Data Availability

The original contributions presented in the study are included in the article/supporting information; further inquiries can be directed to the corresponding author.
